# Systemic above- and belowground cross talk: hormone-based responses triggered by *Heterodera schachtii* and shoot herbivores in *Arabidopsis thaliana*

**DOI:** 10.1093/jxb/erv398

**Published:** 2015-08-31

**Authors:** Nina Kammerhofer, Barbara Egger, Petre Dobrev, Radomira Vankova, Julia Hofmann, Peter Schausberger, Krzysztof Wieczorek

**Affiliations:** ^1^Division of Plant Protection, Department of Crop Sciences, University of Natural Resources and Life Sciences, UFT Tulln, Konrad-Lorenz-Str. 24, 3430 Tulln, Austria; ^2^Institute of Experimental Botany, Academy of Sciences of the Czech Republic, Rozvojová 263, 165 02 Prague 6 - Lysolaje, Czech Republic; ^3^Group of Arthropod Ecology and Behavior, Division of Plant Protection, Department of Crop Sciences, University of Natural Resources and Life Sciences, Peter-Jordan-Str. 82, 1190 Vienna, Austria

**Keywords:** Aboveground–belowground interactions, *Frankliniella occidentalis*, herbivores, *Heterodera schachtii*, phytohormones, plant-parasitic nematodes, systemic responses, *Tetranychus urticae.*

## Abstract

Above- and belowground plant parts are simultaneously attacked by different pests and pathogens. The host mediates these interactions and physiologically reacts, e.g. with local and systemic alterations of endogenous hormone levels coupled with coordinated transcriptional changes. This in turn affects attractiveness and susceptibility of the plant to subsequent attackers. Here, the model plant *Arabidopsis thaliana* is used to study stress hormone-based systemic responses triggered by simultaneous root parasitism by the cyst nematode *Heterodera schachtii* and shoot herbivory by the thrips *Frankliniella occidentalis* and the spider mite *Tetranychus urticae*. First, HPLC/MS and quantitative reverse transcriptase PCR are used to show that nematode parasitism strongly affects stress hormone levels and expression of hormone marker genes in shoots. Previous nematode infection is then demonstrated to affect the behavioural and life history performance of both arthropods. While thrips explicitly avoid nematode-infected plants, spider mites prefer them. In addition, the life history performance of *T. urticae* is significantly enhanced by nematode infection. Finally, systemic changes triggered by shoot-feeding *F. occidentalis* but not *T. urticae* are shown to make the roots more attractive for *H. schachtii*. This work emphasises the importance of above- and belowground signalling and contributes to a better understanding of plant systemic defence mechanisms against plant-parasitic nematodes.

## Highlight

Attack by different below- and aboveground organisms alters the condition of the host plant through systemic changes in phytohormone levels, which modulate defence responses and affect subsequent attackers.

## Introduction

Plants have evolved sophisticated defence strategies to effectively combat attack by different pathogens and herbivorous arthropods. Thus, they perceive an attack and translate this ‘perception’ into an appropriate defensive response. For many years, locally occurring reactions were the focus of various studies (Dangl and Jones, 2001; Dicke and Hilker, 2003; Pieterse and van Loon, 2004). However, in both natural and agricultural ecosystems, attacks by above- or belowground pathogens and herbivores may induce systemic effects in addition to local responses. These can either occur through induction of defence pathways (Ithal *et al.*, 2007; Hamamouch *et al.*, 2011) or through their active suppression by the invader (Glas *et al.*, 2014; Goverse and Smant, 2014; Alba *et al.*, 2015). These multilayered interactions are very likely more important ecologically than the local responses (Groen *et al.*, 2013). Via pathogen-associated molecular patterns (PAMP) or herbivore-associated molecular patterns (HAMP) simultaneous attacks may activate PAMP- or HAMP-triggered immunity (Jones and Dangl, 2006; Acevedo *et al.*, 2015). Both can trigger stress-signalling and systemic resistance phenomena in the whole plant that lead to a number of different defence responses, such as changes in gene expression and in endogenous hormone levels (Choudhary *et al.*, 2007; Fu and Dong, 2013). Indeed, this interplay is fine-tuned by a set of phytohormones, including jasmonic acid (JA), salicylic acid (SA), and ethylene (ET), and is highly species-specific (Pieterse *et al.*, 2009; Kutyniok and Müller, 2012; Groen *et al.*, 2013). In general, it is widely accepted that ET and JA are responsible for defence against herbivores and necrotrophic pathogens (Kessler and Baldwin, 2002; Thaler *et al.*, 2004; Pieterse *et al.*, 2009; Wei *et al.*, 2011; Wei *et al.*, 2014), whereas SA mainly acts against biotrophic pathogens and some phloem-sucking insects, such as whiteflies (Thaler *et al.*, 2004; Spoel *et al.*, 2007; Pieterse *et al.*, 2009; Zhang *et al.*, 2009; Wei *et al.*, 2014). However, there are exceptions to these principles. For instance, the JA pathway is also effective against some biotrophic pathogens (Ellis *et al.*, 2002; Pozo *et al.*, 2004; Thaler *et al.*, 2004). Moreover, recent studies suggest that microorganisms in the saliva of herbivores may suppress the herbivore-typical JA pathway and, instead, elicit the SA pathway that is typical for biotrophic pathogens (Chung *et al.*, 2013).

In case of herbivorous arthropods, the elicitation of plant defence also strongly depends on the type of damage to the plant tissue, which varies widely among species. Chewing insects, such as caterpillars, consume significant portions of plant tissue, whereas phloem-feeding insects like aphids provoke minimal direct damage. Chelicerates, such as the two-spotted spider mite *Tetranychus urticae*, a major pest on many different crop species, pierce parenchyma cells to feed on cell contents (Zhurov *et al.*, 2014). *Frankliniella occidentalis,* the Western flower thrips, another agricultural pest of worldwide relevance, feeds on epidermal and sub-epidermal cells by rasping and sucking (Kirk and Terry, 2003). In general, plant responses to herbivore damage suggest that plants perceive this type of attack by recognizing specific elicitors, which mainly originate from the arthropods’ oral secretions (Bonaventure, 2012) and/or by damage-associated molecular patterns (DAMPs) that result from wounding or enzymatic damage caused by the herbivore (Erb *et al.*, 2012). This includes basal defence responses, in which, besides salicylic acid (SA) and ethylene (ET), jasmonic acid (JA) plays a major role (Zhurov *et al.*, 2014). Accordingly, host preference and herbivore oviposition can be affected either negatively or positively by exogenous JA or methyl jasmonate (mJA) application. JA-treatment of *Brassica oleracea* plants, for instance, resulted in avoidance by *Pieris rapae* and *P. brassicae* but preference by *Plutella xylostella* (Bruinsma *et al.*, 2007). For *F. occidentalis*, some previous findings indicate that both adults and larvae respond negatively to jasmonates (Thaler *et al.*, 2001; Egger and Kochier, 2014). First choice experiments with the spider mite *T. urticae* (Wei *et al.*, 2014) showed that JA application makes plants less attractive for females at earlier time points but more attractive at later time points after hormone treatment. Similarly, SA alone and joint application of SA and JA enhanced the attraction of plants for female mites (Wei *et al.*, 2014).

Several experiments have been conducted to identify systemic effects triggered by simultaneous attack by different pathogens. For instance, an early *Pseudomonas syringae* infection can affect subsequent plant–pathogen and plant–herbivore interactions, either compromising or aiding the response to a second attacker of similar or different identity (Cui *et al.*, 2005; Groen *et al.*, 2013; Fu and Dong, 2013). Cui *et al.* (2002) described *P. syringae* infection of lower rosette leaves triggering a systemic induced susceptibility to herbivorous *Trichoplusia ni* larvae in the upper leaves. Further, the beet armyworm *Spodoptera exigua* showed reduced growth rates when fed on cotton leaves of plants with prior root damage caused by the click beetle *Agriotes lineatus* (Bezemer *et al.*, 2003).

Plant-parasitic nematodes (PPN), such as cyst nematodes (CN) and root-knot nematodes (RKN), are biotrophic pathogens that trigger a wide range of transcriptional and metabolic changes as well as altered levels in endogenous hormones (Alkharouf *et al.*, 2006; Ithal *et al.*, 2007; Puthoff *et al.*, 2007; Szakasits *et al.*, 2009; Hofmann *et al.*, 2010; Mazarei *et al.*, 2011; Cabello *et al.*, 2014; Kammerhofer *et al.*, 2015). Phytohormones in particular play important roles in host responses to the nematode infection (Wubben *et al.*, 2001; Lohar *et al.*, 2004; Wubben *et al.*, 2008; Hamamouch *et al.*, 2011). For instance, auxin (indole-3-acetic acid, IAA) was found to be indispensable for successful syncytium formation (Karczmarek *et al.*, 2004; Wang *et al.*, 2007; Grunewald *et al.*, 2008; Gutjahr and Paszkowski, 2009; Swiecicka *et al.*, 2009). In addition, Wubben *et al.* (2001, 2004) showed ET-overproducing mutants to be hyper-susceptible to *Heterodera schachtii*, whereas ET-insensitive mutants exhibited a lower susceptibility. Active suppression of JA biosynthesis and signalling also plays an important role during syncytium formation (reviewed in Gutjahr and Paszkowski, 2009). Wubben *et al.* (2008) showed that SA adversely affects development of *H. schachtii* females. However, in contrast to these well-described local changes, the systemic effects triggered by nematodes are poorly understood. For instance, Hamamouch *et al.* (2011) found up-regulation of SA marker genes *PR-1* and *PR-5* at 9 days after infection (dai), whereas the JA marker *PR-4* (*HEL*) was down-regulated at 15 dai in shoots. Similarly, Wubben *et al.* (2008) found enhanced *PR-1* transcription at 3 dai and a slight elevation of endogenous SA levels in shoots of infected plants at 4 dai. Therefore, it is assumed that systemic changes in hormone levels triggered by concurrent nematode attack and infestation by shoot attackers have significant impacts on all organisms involved in the above- and belowground sphere of the host. Accordingly, van Dam *et al.* (2005) found that the leaf-feeding larvae of *P. rapae* grow more slowly and are less likely to pupate on black mustard plants (*Brassica nigra*) infested with the root lesion nematode *Pratylenchus penetrans*.

Because insects and PPNs are frequently occurring and very diverse plant-attackers, their interaction mediated by plants is particularly interesting (reviewed in van Dam and Heil, 2011; Johnson *et al.*, 2012; Soler *et al.*, 2013; Wondafrash *et al.*, 2013). Therefore, the aim of this study was to shed more light on induced resistance-related effects triggered by simultaneous root-feeding of the CN *H. schachtii* and shoot-feeding by the herbivores *F. occidentalis* and *T. urticae*. To this end, the effects triggered by nematodes were investigated to determine if they are strong enough to change host attractiveness to shoot herbivores or to modify their behavioural and life history performance (from below- to aboveground) on *Arabidopsis thaliana*. Second, it was determined whether shoot herbivores are triggering systemic effects in roots that change the host attractiveness to nematodes (from above- to belowground). Overall, the findings of this study should significantly contribute to a better understanding of the complex systemic defence mechanisms employed by plants that are simultaneously attacked by different organisms.

## Material and methods

### *A. thaliana* culture

Seeds of *A. thaliana* (Col-0) were surface-sterilized (0.7% NaClO, 40% EtOH) for 8min, washed in 70% EtOH, and immediately rinsed three times in distilled water. Ten seeds per dish (94mm in diameter) were placed on 0.2 Knop medium. Subsequently they were grown in a 16h light/8h dark (L16D8) photoperiod at 23°C for 12 days.

### Nematode culture, inoculation, and attraction assay

*H. schachtii* was obtained from sterile stock culture on *Sinapis alba* cv. Albatros. Second-stage juveniles (J2) were surface sterilized in 0.05% HgCl_2_ for 3min and immediately washed three times in double-distilled H_2_O. Next, 12-day-old *A. thaliana* seedlings were inoculated with 50 J2s directly on the roots (Sijmons *et al.*, 1991). Inoculated plates were kept in the dark for 24h and subsequently transferred into a growing chamber in a L16D8 photoperiod.

The nematode attraction assay was performed according to Dalzell *et al.* (2011). Briefly, 2% water agar plates with cylindrical counting wells (8mm in diameter) connected via a cylindrical channel (20mm × 2.5mm) were prepared. Agar discs containing root exudates, obtained from treated and non-treated *A. thaliana* plants grown on 0.2 Knop media as described above, were placed into the counting wells. One hundred J2s were placed in the middle of the connecting channel. Six plates for each treatment and each replicate were prepared and stored in the dark at room temperature. After 3.5h, the number of J2s that reached either one or the other well was counted and classified as attracted by the root exudate of the respective agar disc. Experiments were performed in three independent replicates with six plates each. Results were calculated as attraction rate (%) of the total number of used nematodes.

### Thrips and mite culture, inoculation, and bioassays

*F. occidentalis* (class Insecta) was reared on detached *Phaseolus vulgaris* leaves on 1% water agar (Agar, Sigma-Aldrich, Vienna, Austria) in Petri dishes in a climate chamber at 24°C in a L16D8 photoperiod (Egger *et al.*, 2014). *T. urticae* (class Arachnida) was reared on whole bean plants at room temperature in a L16D8 photoperiod (Hoffmann *et al.*, 2011). For quantitative reverse transcriptase (qRT)-PCR and HPLC/MS analysis, two adult spider mite females or one adult thrips female was placed on each plant to feed for 24h. Whole shoot and root tissue was sampled and immediately put in liquid nitrogen for subsequent analysis.

*F. occidentalis* and *T. urticae* were further used for choice and no-choice performance assays comparing plants previously infected with *H. schachtii* and uninfected control plants. Plants used for these bioassays were singly grown in 12-well plates on Knop media containing 1% Daichin agar. Inoculation of 12-day-old seedlings was performed with 50 *H. schachtii* J2s. Two agar cylinders were obtained 24h after inoculation (hai) from the 12-well plates, containing either an infected or control plant, and placed next to each other in one petri dish (50mm ø). Subsequently, 10 second-stage thrips larvae (L2) were placed on a piece of filter paper between these two plants and their position was monitored immediately after release—immediate choice indicates the plant first chosen by the L2. The position of the larvae was monitored after 30min and then every hour for 4h in total. For the mite assay, one adult female was placed between the plants and its position on the infected or uninfected plant was monitored as described above.

For the spider mites, an additional no-choice performance assay was performed on single plants grown in agar plates (50mm ø). Here, a single adult female was placed on each plant, either previously infected with *H. schachtii* (24 hai) or uninfected. After 24 and 48h, the following parameters were monitored: presence (yes/no), location (adaxial or abaxial leaf surface), oviposition (number of eggs), and damage (categorized based on discoloration as no, light, or severe damage according to the leaf area visibly sucked by the mites). In case of female disappearance, after 24h a new mite was placed on the plant.

### qRT-PCR analysis

Whole roots or shoots of approximately 20–30 *A. thaliana* seedlings were sampled in three independent biological replicates. RNA was extracted using Qiagen RNA Plant Mini Kit according to the manufacturer’s instructions (Qiagen, Hilden, Germany). RNA was analysed using a Nanodrop 2000c Spectrophotometer (Thermo Scientific, Peqlab, Germany); cDNA synthesis was performed using SuperScriptIII reverse transcriptase (Invitrogen, Carlsbad, CA, USA) according to the manufacturer’s instructions. Reference genes *UBP22* and *18S RNA* (Hofmann and Grundler, 2007) were used for analysis (see Supplementary Table S1 for all primer sequences and Supplementary Table S2 showing the validation of *18S rRNA* and *UBP22* as reference genes). qPCR was performed using ABI PRISM 7300 (Applied BioSystems, Waltham, MA, USA). The final reaction volume was 25 µl containing 12.5 µl SYBR Green reaction kit (Invitrogen), 0.5 µl 10μM primers, 9.5 µl double-distilled H_2_O, and 2 µl of cDNA template. Three independent biological replicates each in technical triplicates (averaged before statistical analysis) were tested. The PCR reaction was conducted as follows: 95°C for 10min, then 40 cycles of 95°C for 15 s and 60°C for 60 s. Changes in transcript levels were calculated using the 2^-∆∆ct^ method (Schmittgen and Livak, 2008).

### Hormone quantification

For hormone quantification, 12-day-old *A. thaliana* plants were infected with nematodes, thrips, or mites as described above. Shoots and roots of approximately 20–40 individual seedlings were pooled and collected 24 hai in four biological replicates, weighed, frozen in liquid nitrogen, and stored at −80°C. Hormones were purified and analysed according to Dobrev and Kaminek (2002) and Dobrev and Vanková (2012). Samples of ~200mg were homogenized and extracted with methanol/water/formic acid (15/4/1, v/v/v). The following labelled internal standards (10 pmol per sample) were added: ^13^C_6_-IAA (Cambridge Isotope Laboratories, Tewksbury, MA, USA), ^2^H_4_-SA (Sigma-Aldrich), ^2^H_2_-GA_4_, ^2^H_2_-GA_19_, ^2^H_6_-ABA, ^2^H_5_-*trans*Z, ^2^H_5_-*trans*ZR, ^2^H_5_-*trans*Z7G, ^2^H_5_-*trans*Z9G, ^2^H_5_-*trans*ZOG, ^2^H_5_-*trans*ZROG, ^2^H_5_-*trans*ZRMP, ^2^H_3_-DHZ, ^2^H_3_-DHZR, ^2^H_3_-DHZ9G, ^2^H_6_-iP, ^2^H_6_-iPR, ^2^H_6_-iP7G, ^2^H_6_-iP9G, and ^2^H_6_-iPRMP (Olchemim, Olomouc, Czech Republic). Extracts were purified using an SPE-C18 column (SepPak-C18, Waters, Milford, MA, USA) and separated on a reverse phase-cation exchange SPE column (Oasis-MCX, Waters, Milford, MA, USA). The first hormone fraction, eluted with methanol, contained abscisic acid (ABA) and other acidic hormones; the second fraction, eluted with 0.35M NH_4_OH in 70% methanol, contained cytokinin metabolites and 1-aminocyclopropane-1-carboxylic acid (ACC). Both fractions were separated by HPLC (Ultimate 3000, Dionex, Sunnyvale, CA, USA) and the hormones were quantified using a hybrid triple quadrupole/linear ion trap mass spectrometer (3200 Q TRAP, Applied Biosystems) operated in selected reaction monitoring mode.

### Statistical analyses

Stat Graphics plus 4.0 software (Statpoint Technologies Inc., Warrenton, VA, USA) was used to analyse the data obtained from qRT-PCR analysis and hormone quantification. A one-way ANOVA (post-hoc Tukey) was performed to determine statistical differences between the variants. Differences in nematode attraction to herbivore-infested plants were analysed using paired *t*-tests. SPSS 21 was used for the thrips and mite bioassays. For the thrips choice assay, generalized estimating equations (GEE) were used to analyse the preference of the larvae (counts of events; binomial distribution with logit link) for nematode-infected and uninfected plants over time (used as inner-subject variable, autocorrelation structure between observations). Similarly, GEE was used for the mite choice assay to analyse the preference of the mites (binomial distribution with logit link) for nematode-infected and uninfected plants over time (used as inner-subject variable, autocorrelation structure between observations). For the mite no-choice assay, GEEs were used to compare female presence (yes/no; binomial distribution with logit link), position (abaxial/adaxial; binomial distribution with logit link), activity (moving/stationary; binomial distribution with logit link), damage caused (Poisson distribution, log link), and the number of eggs laid (Poisson distribution, log link) over time (autocorrelation structure between observations) on nematode-infected and uninfected plants. The number of eggs laid at 24h and 48h was additionally analysed by separate generalized linear models (GLM; Poisson distribution with log link).

## Results

### *H. schachtii* infection modulates endogenous stress hormone levels and transcription of hormone-related genes in shoot

**Fig. 1.**
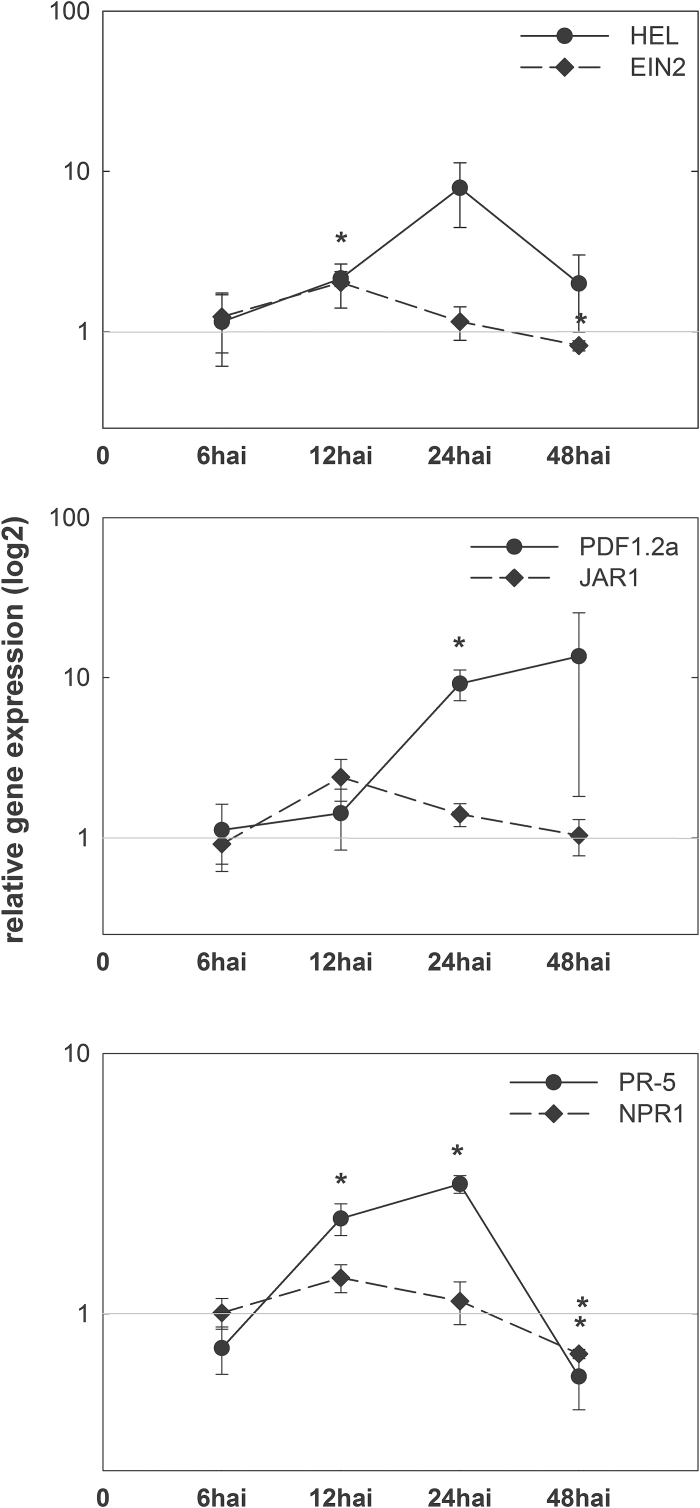
Fold changes (log_2_) of hormone-related marker genes in shoots of *H. schachtii*-infected *A. thaliana* plants at 6, 12, 24, and 48 hai compared to uninfected control plants. Values are means ±SE, n = 3, asterisks indicate significant differences (*P* < 0.05).

To monitor possible systemic effects triggered by root parasitism of *H. schachtii* in *A. thaliana*, a qRT-PCR was performed analysing recently used marker genes for JA, ET, and SA. Previously, *UBP22* and *18S rRNA* transcript levels have been shown to be stable in nematode-infected root tissue (Hofmann and Grundler, 2007). Here, first their stability in shoot tissue was tested by qRT-PCR. This analysis showed that transcript levels of both house-keeping genes in shoots of plants infected with *H. schachtii* were stable and could therefore be used as internal reference genes in the following experiments (Supplementary Table S2). Subsequent qRT-PCR showed a significant up-regulation of the JA/ET-responsive genes *HEL* at 12 hai (*P* = 0.0073) and *PDF1.2a* at 24 hai (*P* = 0.0151). The level of the ET-signalling gene *EIN2* also changed in shoot, exhibiting a slight up-regulation at 12 hai and slight, but significant, down-regulation at 48 hai (*P* = 0.0384). The expression of the JA-signalling gene *JAR1* was not altered at either tested time point. The SA-dependent *PR-5* gene was significantly up-regulated at both 12 hai (*P* = 0.0150) and 24 hai (*P* = 0.0009), whereas at 48 hai it was down-regulated (*P* = 0.0437). *NPR1* did not show any differential regulation until 48 hai, when it was slightly down-regulated (*P* = 0.0005) (Fig. 1).

**Fig. 2.**
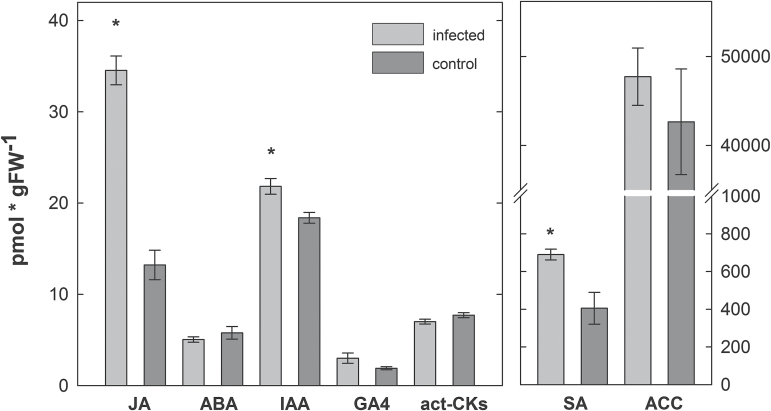
Hormone quantification in shoots of *H. schachtii*-infected *A. thaliana* plants compared to uninfected control plants. Samples were collected at 24 hai. ACC, 1-aminocyclopropane-1-carboxylic acid; ABA, abscisic acid; act-CKs, active cytokinins (*trans*-zeatin, dihydrozeatin, isopentenyladenine, *cis*-zeatin, and the corresponding ribosides); GA4, gibberellin 4;IAA, indole-3-acetic acid; JA, jasmonic acid; SA, salicylic acid. Values are means ±SE, n = 4, asterisks indicate significant differences (*P* < 0.05).

To verify these results, HPLC/MS analyses were conducted. Because the most severe effects on the expression levels of hormone-related genes triggered by nematode infection were found at 24 hai, this time point was used for subsequent hormone quantification. This analysis revealed elevated levels of JA and SA in shoots of nematode-infected plants. The level of ET-precursor ACC was not changed, whereas IAA was present at higher levels in shoots. Similarly, the level of ABA catabolite dihydrophaseic acid (DPA) was elevated (Supplementary Table S3), whereas the level of ABA itself did not change because of nematode root infection (Fig. 2).

### Systemic effects triggered by *H. schachtii* change behaviour and performance of shoot herbivores

**Fig. 3.**
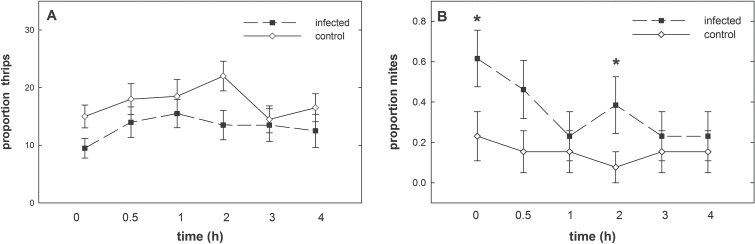
Proportions of (A) *F. occidentalis* (GEE *P* = 0.042) and (B) *T. urticae* (GEE *P* = 0.05) choosing between an *A. thaliana* plant infected by *H. schachtii* (24 hai) and an uninfected control. Thrips L2s were released in groups of 10 (n = 20); adult spider mite females were released singly (n = 13). Values are means ±SE, asterisks indicate significant differences (*P* < 0.05).

Choice assays performed with thrips L2 revealed a significant preference for the uninfected control over the plants infected with *H. schachtii* (Fig. 3A; GEE: Wald ӽ_1_^2^ = 4.149, *P* = 0.042). This preference did not statistically change over time, although a slight reduction was observed (Wald ӽ_10_^2^ = 15.766, *P* = 0.107). In contrast to thrips larvae, the spider mite females preferred plants infected with *H. schachtii* over the uninfected control plants (Fig. 3B; GEE: Wald ӽ_1_^2^ = 3.725, *P* = 0.05). This preference was the strongest immediately after release and weakened over time (Wald ӽ_5_^2^ = 230.117, *P* < 0.001).

**Fig. 4.**
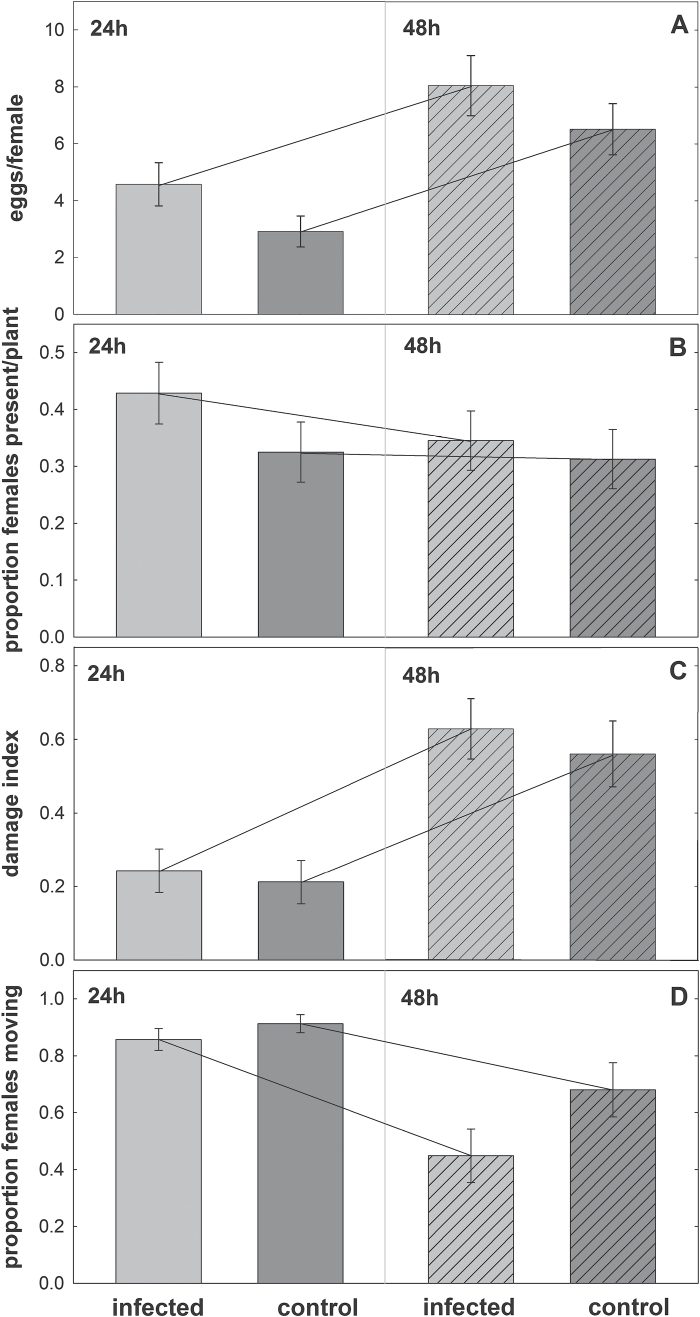
Spider mite no-choice performance assay. Single adult females were released on *A. thaliana* plant infected with *H. schachtii* (24 hai; n = 84) or uninfected control plants (n = 80) and monitored after 24 and 48h. Values are means ±SE.

**Fig. 5.**
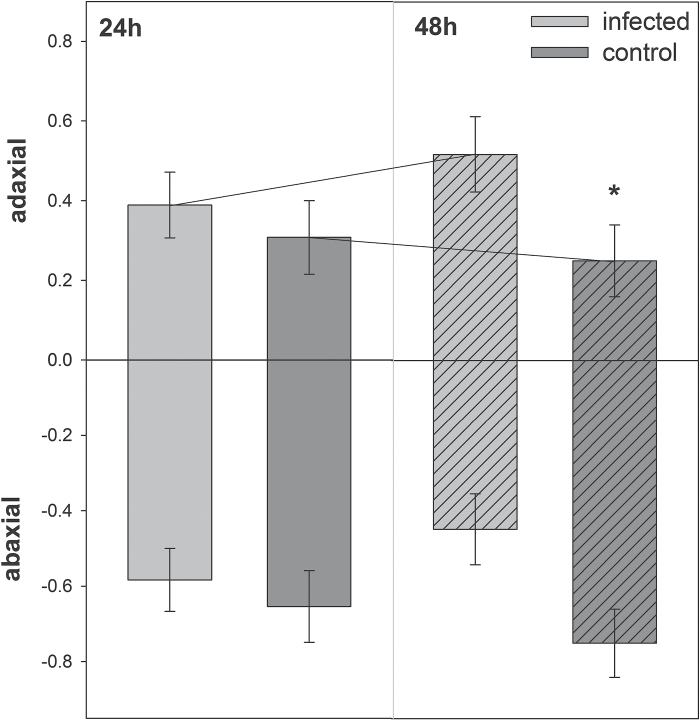
Proportion of spider mite females on the abaxial and adaxial leaf surfaces of *A. thaliana* plants infected with *H. schachtii*. Single adult females were released on nematode-infected (24 hai; n = 84) or uninfected control (n = 80) plants and their position on the leaves was monitored after 24h and 48h. Values are means ±SE.

In the no-choice performance assay, the *T. urticae* females performed better on nematode-infected than uninfected plants. They were less active, caused more damage, tended to deposit more eggs, and had a stronger preference for the abaxial leaf surface on nematode-infected plants as compared to the uninfected control (Figs. 4 and 5, Table 1). Although oviposition across time was insignificant, time-dependent oviposition differed (Table 1), with females depositing more eggs on infected than uninfected plants at 24h (GLM: Wald ӽ_1_^2^ = 3.725, *P* = 0.077) but not at 48h (Wald ӽ_1_^2^ = 1.217, *P* = 0.270).

**Table 1.** Results of generalized estimating equations for the performance of the spider mite *T. urticae* on nematode-infected and uninfected *A. thaliana* plants over time

	Infected/uninfected		Infected/uninfected (time)	
	Wald ӽ^2^	*P*-value	Wald ӽ^2^	*P*-value
Eggs	2.380	0.123	77.947	<0.001
Presence	1.056	0.304	2.678	0.263
Damage	4.793	0.632	1.469	<0.001
Position	0.230	0.029	54.748	0.480
Activity	5.563	0.018	24.419	<0.001

### Shoot herbivory triggers effects in systemic root

**Fig. 6.**
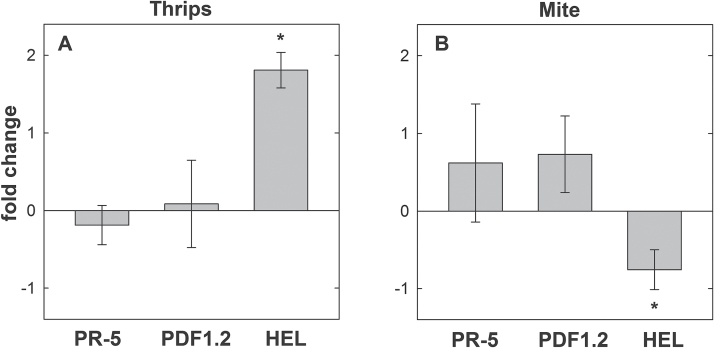
Fold changes (∆∆ct) of hormone-related marker genes in roots of *A. thaliana* plants infested by (A) *F. occidentalis* or (B) *T. urticae* compared to non-infested control plants (n = 3 for each herbivore). Values are means ±SE, asterisks indicate significant differences (*P* < 0.05).

The expression of hormone-related marker genes differed between plants invaded by the two different arthropods. Feeding of *F. occidentalis* triggered a significant up-regulation of ET/JA-marker gene *HEL* in systemic roots. The SA-marker *PR-5* and ET/JA-marker *PDF1.2* did not show any changes. In contrast, feeding by *T. urticae* resulted in a significant down-regulation of *HEL* but did not cause any differential regulation of *PR-5* or *PDF1.2* (Fig. 6).

**Fig. 7.**
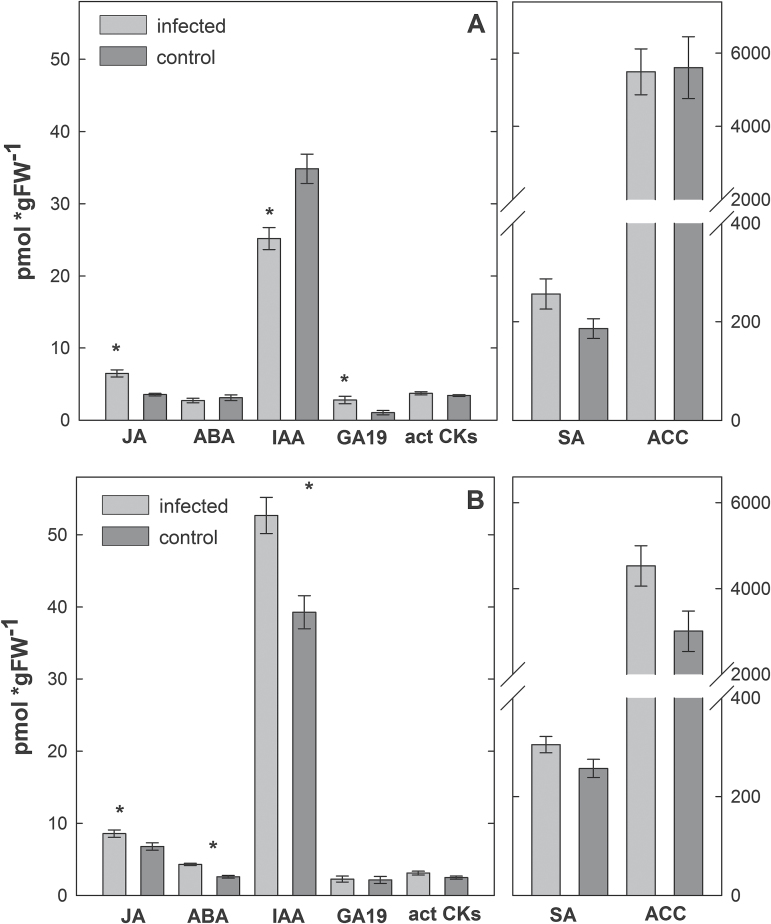
Hormone quantification in roots of (A) thrips-infested and (B) spider mite-infested compared to non-infested *A. thaliana* plants. Samples were collected at 24 hai. ACC, 1-aminocyclopropane-1-carboxylic acid; ABA, abscisic acid; act-CKs, active cytokinins (*trans*-zeatin, dihydrozeatin, isopentenyladenine, *cis*-zeatin, and the corresponding ribosides); GA_19_, gibberellin 19;IAA, indole-3-acetic acid; JA, jasmonic acid; SA, salicylic acid. Values are means ±SE, n = 4, asterisks indicate significant differences *(P <* 0.05).

Hormone quantification revealed a significant elevation of endogenous ABA and IAA levels in systemic roots of plants infested with spider mites; only a mild increase was observed in the case of active cytokinins (Fig. 7). Spider mite feeding did not significantly affect the concentration of the precursor of active gibberellins (GA_19_). In contrast, in roots of plants infested with thrips, IAA concentration was decreased, GA_19_ was elevated, and active cytokinin and ABA levels did not change. However, the concentration of the ABA catabolite DPA was elevated (Supplementary Table S4). Both herbivores significantly elevated JA levels in systemic root and did not change levels of SA or the ET precursor ACC.

### Systemic effects triggered by shoot herbivory change root attractiveness towards *H. schachtii*

**Fig. 8.**
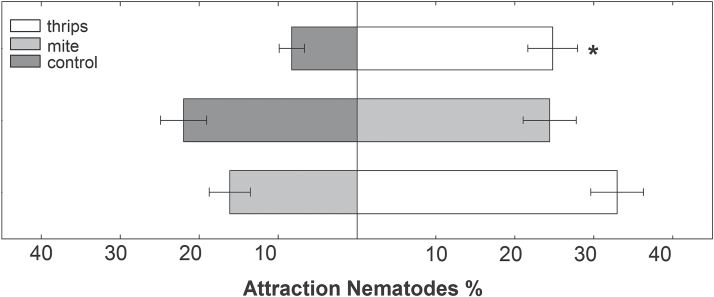
Nematode attraction assays illustrating movement of 100 *H. schachtii* J2s towards agar discs containing root exudates of previously thrips- or spider mite-infested *A. thaliana* plants (24 hai) and non-infested control plants. Values are means ±SE, n = 18, asterisks indicate significant differences (paired, *P* < 0.05).

Possible effects triggered by shoot herbivory were tested in attraction assays analysing the attractiveness of plants previously infested with herbivores to J2s of *H. schachtii*. Agar discs containing root exudates from plants infested with either *F. occidentalis* or *T. urticae* were used for this analysis. Results show that nematodes were more attracted to root exudates from plants previously infested by thrips (*P* ≤ 0.001). In contrast, plants infested with spider mites did not show any difference in their attractiveness to nematodes in comparison to control (*P* = 0.6627). Further, when directly comparing thrips- and mite-infested plants, the exudates from plants fed on by thrips were slightly more attractive for *H. schachtii* juveniles (*P* = 0.0997), although this was not statistically significant (Fig. 8).

## Discussion

Most studies available to date have focused on the plant defence above- or belowground activated as a response to pathogen or herbivore attack. However, substantially less knowledge is available on systemic effects triggered by simultaneous attack by different pests above- and belowground. Cross talk between these two different spheres of the host plant is ecologically important and has profound effects on natural and agricultural food webs (Erb *et al.*, 2008; Groen *et al.*, 2013). The interactions between belowground PPN and aboveground herbivorous arthropods are especially crucial because of their ubiquitous occurrence (reviewed in van Dam and Heil, 2011; Johnson *et al.*, 2012; Soler *et al.*, 2013; Wondafrash *et al.*, 2013). The results presented here provide novel insights into systemic responses triggered in the model plant *A. thaliana* by concomitant root infection by the CN *H. schachtii* and shoot infestation by two different herbivorous arthropods, two-spotted spider mites *T. urticae* and Wester flower thrips *F. occidentalis*. The way in which these different attackers induce hormone-based systemic responses are examined, as well as host susceptibility, and how they influence each other’s behavioural and life history performance.

### *H. schachtii* root infection triggers hormone-related systemic responses in shoots

Nematodes induce severe alterations in affected root tissue during the infection process and further development, including changes in expression of genes involved in defence responses, metabolism, nutrient allocation, as well as phytohormone biosynthesis and signalling (Hofmann *et al.*, 2010; Ithal *et al.*, 2007; Alkharouf *et al.*, 2006; Mazarei *et al.*, 2011; Kammerhofer *et al.*, 2015). These modifications have been intensively studied locally within the host root, but some studies have shown that systemic effects in aboveground tissue can mostly be attributed to changed levels of several phytohormones, such as JA, SA, and IAA, and the expression of hormone-related genes (Wubben *et al.*, 2008; Hamamouch *et al.*, 2011; Kyndt *et al.*, 2012).

JA is mostly referred to as being active against necrotrophic pathogens and herbivores (Pieterse *et al.*, 2009) and is often considered as a key component of the central platform triggering systemic plant responses (Farmer and Ryan, 1992; Erb *et al.*, 2008). Moreover, several studies have also indicated that JA plays a crucial role during attack by biotrophic pathogens (Baldwin *et al.*, 1996; van Dam *et al.*, 2001; van Dam *et al.*, 2005; Hamamouch *et al.*, 2011; Kammerhofer *et al.*, 2015). For instance, Hamamouch *et al.* (2011) analysed the transcript levels of several hormone- and defence-related genes in systemic *A. thaliana* shoots at later time points of *H. schachtii* infection. The authors detected a significant up-regulation of a JA-dependent gene, *PR-3,* at 5 and 9 dai, and a down-regulation of *PR-4* (*HEL*) at 14 dai in shoots, although neither gene was regulated locally in roots at tested stages of infection. Similarly, the presented data show a clear up-regulation of *PR-4* (*HEL*), starting at 12 hai, as well as *PDF1.2* at 24 hai, followed by reduced expression at 48 hai. These results are in line with the strongly elevated endogenous JA levels measured in shoots, and are similar to root tissue data recently obtained for similar early stages of nematode infection (Kammerhofer *et al.*, 2015). Interestingly, contrasting results were recently published for RKN infection. Kyndt *et al.* (2012) reported systemic changes in expression of defence-related genes in rice (*Oryza sativa*) infected by *Meloidogyne graminicola*. The authors found decreased expression of genes related to JA biosynthesis at 3 dai and suggested that RKN might suppress the systemic defence in shoots already at early stages of infection. This also indicates that the JA-related systemic responses vary considerably, being highly nematode species- and host-specific. Here, for CN, the JA-dependent defence pathways were induced during early stages of infection not only in nematode-infected root tissue, as previously shown (Kammerhofer *et al.*, 2015), but also exerted systemic effects on shoot tissue, where these responses might be subsequently suppressed as soon as the infection is successfully completed and stable.

In contrast to JA, SA is known to be active against mainly biotrophic pathogens (Pieterse *et al.*, 2009) but can also be induced by some herbivores, e.g., *Bemisia tabaci* (Zarate *et al.*, 2007). For CN, recent research found no changes in endogenous SA levels or expression of SA-marker *PR-5* during early *H. schachtii* root infection (Kammerhofer *et al.*, 2015). However, significantly elevated endogenous levels of SA (24 hai) as well as increased *PR-5* expression at 12 hai and 24 hai were found in this study in shoots. These results demonstrate that SA-dependent defence is already systemic in shoot tissue during early phases of CN parasitism. At later time points, Wubben *et al.* (2008) found a slight but not significant systemic elevation of endogenous SA levels in shoots and enhanced transcript levels for *PR-1*. Similarly, at 9 dai, Hamamouch *et al.* (2011) detected up-regulation of *PR-1*, *PR-2*, and *PR-5* in shoots. These studies, together with results presented here, show that root parasitism by CN triggers durable induction of SA-dependent resistance in shoots. For RKN, a constant down-regulation of SA-responsive genes has been shown in shoots of *A. thaliana* (Hamamouch *et al.*, 2011) and rice (Kyndt *et al.*, 2012). This indicates that the role of SA in shoots fundamentally differs between CN and RKN parasitism.

In contrast to JA and SA, which function mainly as coordinators of plant defence responses, auxins are mainly attributed to regulation of plant growth and development. However, they are now also considered by some authors to be essential factors in defence responses to biotic stress (Mauch-Mani and Mauch, 2005; Fu and Wang, 2011) and in modulation of the feedback between above- and belowground plant parts (Erb *et al.*, 2008). The elevated auxin levels in shoot tissue of nematode-infected plants observed here are in accordance with compensatory shoot growth after root attack by two herbivorous insects (Steinger and Müller-Schärer, 1992) and the up-regulation of an auxin marker gene in *Zea mays* shoots after root damage by *Diabrotica virgifera* (Erb *et al.*, 2008).

Data obtained here for JA, SA, and IAA demonstrate that *H. schachtii* infection causes explicit systemic changes in stress hormone-based defence in shoot tissue, which significantly differ from those locally triggered in the infected roots. These effects essentially change plant responses to following shoot attackers, either in a positive or negative way.

### Systemic responses triggered by *H. schachtii* modulate life history and behavioural performance of herbivores

Recently, growing interest has been dedicated to systemic defence induced by attackers simultaneously infesting different parts of the host plant. A negative influence on performance of shoot herbivores triggered by root-feeding insects or nematodes has previously been reported. For example, Bezemer *et al.* (2003) provided evidence that *Agriotes lineatus*, a root feeder, reduces the performance of the foliar-feeding insect *Spodoptera exigua*. For PPN, van Dam *et al.* (2005) found a decrease in performance of *Pieris rapae* larvae, which grew more slowly and pupated less on plants infected with the endoparasitic nematode *Pratylenchus penetrans*. For RKN, infection of certain chickpea cultivars can lead to breakdown of resistance against specific strains of *Fusarium oxysporum* (Castillo *et al.*, 2003; Palomares-Rius *et al.*, 2011). Other studies found that nematode root infection may negatively affect phloem-sucking aphids (Bezemer and van Dam, 2005; Wurst and van der Putten 2007; Kaplan *et al.*, 2009; Hol *et al.*, 2010). For example, ectoparasitic and migratory endoparasitic nematodes caused fecundity reduction in the aphid *Rhopalosiphum padi* on *Agrostis capillaris* and *Anthoxanthum odoratum* (Bezemer *et al.*, 2005). Kaplan *et al.* (2011) demonstrated that *Meloidogyne incognita* parasitism in roots of *Nicotiana tabacum* causes severe declines in growth and fecundity of aphids. A study conducted with the CN *H. glycines* concluded that aphids prefer uninfected over infected soybean plants, whereupon the aphid behaviour rather than their life history is influenced (Hong *et al.*, 2010). However, the influence of soil nematodes on shoot-feeding insects is rather variable (reviewed in Wondafrash *et al.*, 2013). A number of studies reported positive (Kaplan *et al.*, 2008*a*) and neutral effects of nematode parasitism on the performance of shoot-feeders (Kabouw *et al.*, 2011; Kutyniok and Müller, 2012; Wurst and van der Putten, 2007), indicating that the triggered responses are highly species-specific and also depend, among other factors, on the mode of feeding.

This study examined the way two shoot herbivores, an insect and a mite, with different feeding modes were affected by previous nematode root parasitism. The elevated levels of endogenous JA, SA, and IAA as well as the up-regulation of *PDF1.2*, *HEL*, and *PR-5* in shoots of nematode-infected plants strongly suggests altered susceptibility and/or attraction of these plants to shoot invaders. Indeed, early *H. schachtii* infection explicitly changed the behavioural and life history performance of following shoot herbivores. Attractiveness of the shoots of nematode-infected plants to L2 of *F. occidentalis* was significantly reduced. Similarly, Abe *et al.* (2008, 2009) showed that thrips feeding induces JA-regulated plant defence, which negatively affects thrips oviposition and population density. These authors also showed that exogenous application of JA and mJA onto the leaf surface significantly reduces attraction and settling of *F. occidentalis.* Significantly lower numbers of thrips were found on JA-sprayed plants in the field (Thaler *et al.*, 2001) and jasmonate-baited traps did not attract *F. occidentalis* (James, 2005). Most of these studies focused on the adult stage, but recently Egger and Koschier (2014) found that the larval stage may be similarly deterred by exogenous JA application. De Puysseleyr *et al.* (2011) detected higher susceptibility of the tomato mutant *def1*, which exhibits reduced JA-related defence responses, to *F. occidentalis*, and thus suggested that JA-regulated defence mechanisms restrict thrips damage. Accordingly, it is proposed that the deterrent effect of shoots of nematode-infected plants against thrips might be due, at least in part, to the elevated JA levels and enhanced JA-related defence in aboveground tissue, both of which are triggered belowground by *H. schachtii* root infection.

With regard to the spider mites, Bonte *et al.* (2010) reported reduced fecundity on *Phaseolus vulgaris* plants infected with the ectoparasitic nematodes *Pratylenchus penetrans*. However, CN infection in the presented study had an entirely opposite effect, which is also contrary to the results obtained for thrips. In the choice assays, adult females of *T. urticae* immediately chose the nematode-infected over the control plants. Additionally, subsequent behaviours such as their position on the abaxial and adaxial leaf surface as well as oviposition, damage inflicted, and activity were significantly altered. These results indicate that CN triggered substantial changes within the plant at the onset of parasitism, altering the performance of the mites. These effects declined over time. The most profound differences in mite performance were observed between infected and uninfected plants at the beginning of the bioassay. Wei *et al.* (2014) reported on a similar time-dependent response to JA application. The authors first observed that *T. urticae* showed avoidance behaviour, and then, at later time points, demonstrated an attraction towards treated plants. For SA treatments, they did not find avoidance effects, but observed an attraction later on. Concomitant application of JA and SA resulted in attraction of the mites 72h after hormone application.

The observations from the presented study are in line with several reports showing that root herbivory may enhance the performance of shoot herbivores (Kaplan *et al.*, 2008*a*: Gange and Brown, 1989; Masters and Brown, 1992). Previous studies suggested that this effect might be due to stress-induced allocation and accumulation of amino acids and carbohydrates in leaves (Kaplan *et al.*, 2008*b*; White, 1984; Chapin, 1991). Therefore, it is suggested that the concomitant elevation of JA and SA in *A. thaliana* plants infected with *H. schachtii* as well as possible nutrient allocation shifts resulted in greater attractiveness, as well as improved quality as a host, to *T. urticae* females, whereas thrips were repelled by enhanced JA-regulated defence after CN infection. These findings underscore the complexity of the plant hormone-based mechanisms in the interaction between plant, nematodes, and different herbivores. Which substances or specific signalling pathways are responsible for these opposite results, and whether microorganisms in the saliva of the studied herbivores play a role in defence induction (Chung *et al.*, 2013), needs further scrutiny.

### Shoot herbivory triggers hormone-based systemic responses in roots and changes their attractiveness to *H. schachtii*

To date, far less information is available on the effects induced by aboveground herbivory on belowground pests, especially on PPN (reviewed in Wondafrash *et al.*, 2013). This is presumably because the effects on belowground organisms are difficult to measure owing to their inaccessibility (Bruce and Pickett, 2007). However, there are strong indications that such interactions occur and have substantial effects on subsequent root attackers. The presence of shoot-feeders may either reduce (Moran and Whitham, 1990; Salt *et al.*, 1996; Kaplan *et al.*, 2009) or increase (Russin *et al.*, 1989; Alston *et al.*, 1993; Russin *et al.*, 1993; Kaplan *et al.*, 2009) or have no effects on the performance of belowground herbivores (Masters *et al.*, 1993; Kaplan *et al.*, 2011). This influence varies between nematode species and is at least partly determined by the feeding habits of the insects. For instance, Kaplan *et al.* (2011) found no reciprocal effects of aphid herbivory on performance of the RKN *Meloidogyne incognita.* In contrast, Kaplan *et al.* (2009) showed that mutually positive interactions take place between nematodes and chewing insects (e.g., caterpillars), whereas negative interactions occur with phloem sap-feeders (e.g., aphids). Kutyniok and Müller (2013) found deviating effects of aphid shoot-feeding on *H. schachtii*. At low nitrate supply, aphids had a promoting effect on the nematodes, whereas at high nitrate fertilization they reduced the nematode infestation compared to control plants. For chewing insects, increased numbers of *H. glycines* and *M. incognita* were found on *Glycine max* infested with two different caterpillars (Russin *et al.*, 1989; Alston *et al.*, 1993; Russin *et al.*, 1993). The effects triggered by shoot-feeding insects on performance of nematodes might be due to sink competition (Larson and Whitham, 1997); re-allocation of primary metabolites, such as nitrogen and carbohydrates (Masters *et al.*, 1993); or elevated concentrations of secondary metabolites, such as nicotine or proteinase inhibitors in *Nicotiana attenuata* (Baldwin *et al.*, 1994), reducing survival or reproduction of herbivores.

In this study, shoot-feeding by two different herbivores changed the phytohormone homeostasis of the host, affecting the performance of *H. schachtii*. Feeding by thrips *F. occidentalis*, which damage the epidermal layer with their rasping-sucking mouthparts, resulted in significant up-regulation of both ET- and JA-dependent marker genes *HEL* and *PDF1*.*2* in damaged shoot tissue. In comparison, the systemic effects in roots manifested only in up-regulation of *HEL*. These changes in marker gene expression corresponded to systemic changes in hormone levels in the root tissue. Thus, concentration of JA and its active conjugate JA-Ileu was elevated, whereas ACC and SA levels did not change significantly. Additionally, the ABA catabolite DPA and gibberellin GA_19_ were elevated. IAA and its precursor IAN were reduced compared to non-infested control roots. In contrast, feeding by the spider mite *T. urticae* did not change the hormone marker genes in damaged shoots, whereas in roots *HEL* was down- and *PDF1*.*2* slightly up-regulated (Kammerhofer *et al.*, 2015). It triggered, however, an elevation of JA and the receptor interacting conjugate as well as ABA and auxin in roots.

This study demonstrated that shoot-herbivory by thrips and spider mites causes different hormone-based systemic effects in root tissue. To check whether these changes manipulate root susceptibility towards nematodes, an attraction assay was performed to test the attractiveness of root exudates towards *H. schachtii* collected from plants infested with both herbivores. Only previous shoot-feeding by *F. occidentalis* but not *T. urticae* strongly increased the attractiveness of these plants to the nematodes. These herbivore-specific differences might be, at least partially, due to highly variable changes in hormone levels and defence pathways. It can only be speculated that, for instance, the elevated IAA levels in roots of plants infested with spider mites could have a negative effect, whereas the reduced IAA levels in roots of plants infested with thrips could have a positive effect on nematode attraction. The elevated gibberellin levels in roots of thrips-infested plants could be an indicator for a suitable host for the nematodes.

A number of different stress hormone-based systemic effects triggered in *A. thaliana* shoots by *H. schachtii* root infection have been demonstrated here. These responses significantly differ from those occurring locally in root tissue, which have recently been reported. Nematode infection significantly changed the host plant susceptibility to shoot herbivores, such as thrips and spider mites, modulating their behavioural and life history performance. Additionally, similar hormone-based systemic effects occurred in the opposite direction, from above- to belowground parts of the plant, significantly changing the host susceptibility towards *H. schachtii*. The observed effects and hormone-dependent pathways are highly complex and species-specific. Thus, additional and more comprehensive studies are needed to elucidate these plant-mediated interactions, especially with the aid of specific hormone-related *A. thaliana* mutants. Nonetheless, these results greatly contribute to a better understanding of how plants integrate the induced responses triggered by different plant attackers above- and belowground.

## Supplementary data

Supplementary materials are available at *JXB* online.

Supplementary Table S1. Primers for hormone- and defence marker genes used in qRT-PCR analysis.

Supplementary Table 2. Validation of *18S rRNA* and *UBP22* for their suitability as reference genes for qRT-PCR analysis.

Supplementary Table S3. Hormone quantification of shoots of *A. thaliana* plants infected with *H. schachtii*.

Supplementary Table S4. Hormone quantification of roots of *A. thaliana* plants infested with *F. occidentalis* and *T. urticae* in comparison to roots of non-infested control plants.

Supplementary Data
