# Supplementary material for: Systemic above- and belowground cross talk: hormone-based responses triggered by Heterodera schachtii and shoot herbivores in Arabidopsis thaliana
Source: J Exp Bot. 2015 Aug 31;66(22):7005–17. doi: 10.1093/jxb/erv398 (PMC4765779; doi:10.1093/jxb/erv398)
Supplement: Supplementary Data [file supp_66_22_7005__index.html]

Systemic above- and belowground cross talk: hormone-based responses triggered by Heterodera schachtii and shoot herbivores in Arabidopsis thaliana — Systemic above- and belowground cross talk: hormone-based responses triggered by Heterodera schachtii and shoot herbivores in Arabidopsis thaliana — Supplementary Data 

# Systemic above- and belowground cross talk: hormone-based responses triggered by *Heterodera schachtii* and shoot herbivores in *Arabidopsis thaliana*

## Supplementary Data

Data files

- Supplementary Data - Supplementary Data
