# Supplementary material for: Systemic above- and belowground cross talk: hormone-based responses triggered by Heterodera schachtii and shoot herbivores in Arabidopsis thaliana
Source: J Exp Bot. 2015 Aug 31;66(22):7005–17. doi: 10.1093/jxb/erv398 (PMC4765779; doi:10.1093/jxb/erv398)
Supplement: Supplementary Data [file supp_erv398_jexbot153130_file001.pdf]

Supplementary Tables

Supplementary Table 1. Primers for hormone- and defence marker genes used in qRT-PCR analysis

Gene	Acc. number	Primer sequence
HEL	At3g04720	F: GATAAGCCGTACGCATGGC<br>R: TCACCCTTAAACACTTGCCG
PDF1.2	At5g44420	F: TGTTCTCTTTGCTGCTTTCG<br>R: TTTCCGCAAACCCCTGAC
PR5	At1g75040	F: AGGCTGCAACTTTGACGC<br>R: AGAAATCTTTGCCGCCATC
UBP22	At5g10790	F: ACAACATATGACCCGTTTATCGA<br>R: TGTTTAGGCGGAACGGATACT
18S RNA		F: TGACACGGGGAGGTAGTGACA<br>R: AGTCTGGTAATTGGAATGAGTACAATCTAA
JAR1	At2g46370	F: GCTACATTTGCTGTGATTCCG<br>R: GGTATCGATACAACCCTGCG
NPR1	At1g64280	F: GAGTTGCACTTGCTCAACGTC<br>R: GCTATCTTTACACCCGGTGATG
EIN2	At5g03280	F: CCTTGTCACTAATGGAGCAGG<br>R: CACGATGAAGCCAAGCG

Supplementary Table 2. Validation of *18S rRNA* and *UBP22* for their suitability as reference genes for qRT-PCR analysis. Ct-values obtained for infected vs. non-infected *A. thaliana* tissue. i=infected, ni=non-infected

***H. schachtii* - shoot**

	Mean Ct	SE	p-value
**18S i**	14.37	+/- 0.03	
**18S ni**	14.57	+/- 0.10	0.1986
**UBP22 i**	26.79	+/- 0.21	
**UBP22 ni**	26.95	+/- 0.15	0.5655

***F. occidentalis* - root**

	Mean Ct	SE	p-value
**18S i**	14.53	+/- 0.08	
**18S ni**	14.53	+/- 0.03	0.9450
**UBP22 i**	28.73	+/- 0.38	
**UBP22 ni**	29.19	+/- 0.37	0.4300

***T. urticae* - root**

	Mean Ct	SE	p-value
**18S i**	14.578	+/- 0.07	
**18S ni**	14.5343	+/- 0.03	0.6337
**UBP22 i**	29.54	+/- 0.37	
**UBP22 ni**	29.20	+/- 0.37	0.5479

Supplementary Table 3. Hormone quantification of shoots of *A. thaliana* plants infected with *H. schachtii* in comparison to shoots of non-infected control plants; ABA =abscicic acid, DPA=dihydrophaseic acid, PA=phaseic acid, ABA-GE=abscisic acid glucosyl ester, NeoPA= neophaseic acid, IAA=indole-3-acetic acid, IAA-Asp=indole-3-acetyl-aspartate, OxIAA=2-oxindole-3-acetate, PAA=2-phenylacetic acid, IPya= indole-3-pyruvic acid, IAN= indole-3-acetonitrile, SA=salicylic acid, JA=jasmonic acid, JA-Ileu=jasmonic acid isoleucine, cis-OPDA=12-oxophytodienoic acid, GA=gibberellin, CK=cytokinins, act-CKs=active cytokinins (*trans*-zeatin, dihydrozeatin, isopentenyladenine, *cis*-zeatin and the corresponding ribosides), CK N-glc=cytokinin N-glucosides, CK O-glc=cytokinin O-glucosides,CK P=cytokinin precursors, cZ der= derivatives of *cis*-zeatin, ACC=1-aminocyclopropane-1-carboxylic acid

	**inf.**		**non-inf.**		**statistics**
	**MW**	**SE**	**MW**	**SE**	**p-value**
**ABA**	5.04	0.30	5.78	0.69	0.3639
**DPA**	4.55	0.47	1.73	0.27	0.0020
**PA**	2.54	0.27	2.02	0.27	0.2247
**ABA-GE**	15.54	1.56	13.04	1.51	0.2933
**NeoPA**	0.11	0.02	0.14	0.08	0.7259
**IAA**	21.83	0.87	18.39	0.59	0.0171
**IAA-Asp**	13.57	1.69	11.67	0.60	0.3306
**OxIAA**	235.55	19.99	218.88	20.10	0.5780
**PAA**	268.36	31.28	237.26	21.12	0.4415
**IPyA**	32.12	3.77	17.69	3.15	0.0260
**IAN**	896.41	28.63	775.06	73.50	0.1748
**SA**	690.39	28.87	405.46	84.18	0.0186
**JA**	34.54	1.58	13.22	1.61	0.0001
**JA-Ileu**	0.18	0.09	0.09	0.02	0.3559
**cisOPDA**	1206.91	326.06	867.88	40.06	0.3419
**GA_4**	3.01	0.56	1.91	0.17	0.1106
**GA_8**	0.80	0.21	0.81	0.18	0.9803
**GA_{19}**	4.49	0.56	4.98	0.39	0.4992
**act CK**	7.01	0.26	7.72	0.28	0.1132
**CK N-glc**	53.78	3.42	55.46	1.71	0.6767
**CK O-glc**	3.95	0.27	3.46	0.40	0.3459
**CK P**	8.23	0.25	8.32	0.24	0.8167
**cZ der**	6.60	0.40	7.59	0.16	0.0617
**ACC**	47725.97	3222.76	42652.97	5933.27	0.4809

Supplementary Table 4. Hormone quantification of roots of *A. thaliana* plants infested with *F. occidentalis* and *T. urticae* in comparison to roots of non-infested control plants; ABA=abscicic acid, DPA=dihydrophaseic acid, PA=phaseic acid, IAA=Indole-3-acetic acid, IAN= indole-3-acetonitrile, SA=salicylic acid, JA=jasmonic acid, JA-Ileu=jasmonic acid isoleucine, cis-OPDA=12-oxophytodienoic acid, GA=gibberellin, ACC=1-aminocyclopropane-1-carboxylic acid

**THRIPS ROOTs**	**infested**	**control**	**p-value**
ABA	2.72	3.10	0.4798
DPA	20.24	11.18	0.0160
PA	0.81	0.89	0.8164
IAA	25.19	34.84	0.0052
IAN	474.25	1226.25	0.0000
SA	256.76	186.09	0.0897
JA	6.48	3.54	0.0005
JA-Ileu	0.70	0.14	0.0186
cisOPDA	350.39	276.26	0.0838
GA_4	0.20	0.32	0.4869
GA_8	0.78	1.15	0.1950
GA_{19}	2.79	1.04	0.0202
act CK	3.72	3.41	0.2777
ACC	5490.72	5603.34	0.9173

**MITE ROOTs**	**infested**	**control**	**p-value**
ABA	4.30	2.59	0.0001
DPA	26.48	15.28	0.0007
PA	0.87	0.64	0.2729
IAA	52.68	39.28	0.0043
IAN	1052.06	722.23	0.0450
SA	305.36	257.00	0.0882
JA	8.56	6.78	0.0377
JA-Ileu	0.45	0.25	0.5134
cisOPDA	500.63	368.46	0.0096
GA_4	0.70	0.35	0.1379
GA_8	1.23	0.67	0.1548
GA_{19}	2.27	2.15	0.8523
act CK	3.10	2.48	0.1037
ACC	4528.48	3006.43	0.0516
